# Applied K Fertilizer Use Efficiency in Pineapples Grown on a Tropical Peat Soil Under Residues Removal

**DOI:** 10.1100/tsw.2005.9

**Published:** 2005-01-21

**Authors:** Osumanu H. Ahmed, Husni M. H. Ahmad, Hanafi M. Musa, Anuar A. Rahim, Syed Omar S. Rastan

**Affiliations:** Department of Land Management, Faculty of Agriculture, Universiti Putra Malaysia, 43400 UPM Serdang, Selangor, Malaysia

**Keywords:** potassium fertilizers, pineapples, tropical peat soils, residues, leaching

## Abstract

In Malaysia, pineapples are grown on peat soils, but most K fertilizer recommendations do not take into account K loss through leaching. The objective of this study was to determine applied K use efficiency under a conventionally recommended fertilization regime in pineapple cultivation with residues removal. Results showed that K recovery from applied K fertilizer in pineapple cultivation on tropical peat soil was low, estimated at 28%. At a depth of 0–10 cm, there was a sharp decrease of soil total K, exchangeable K, and soil solution K days after planting (DAP) for plots with K fertilizer. This decline continued until the end of the study. Soil total, exchangeable, and solution K at the end of the study were generally lower than prior values before the study. There was no significant accumulation of K at depths of 10–25 and 25–45 cm. However, K concentrations throughout the study period were generally lower or equal to their initial status in the soil indicating leaching of the applied K and partly explained the low K recovery. Potassium losses through leaching in pineapple cultivation on tropical peat soils need to be considered in fertilizer recommendations for efficient recovery of applied K.

## INTRODUCTION

Large-scale production of pineapples on tropical peat soils is characterized by *in situ* burning of crop residues before subsequent replanting. Following the 1997/1998 economic loss in the agricultural sector due to fires and haze in South-East Asia[1,2,3], *in situ* burning of crops residues such as pineapple residues is being discouraged. Studies on product development from pineapple residues have shown some impressive and promising results[4,5,6], but the existing K fertilization program for this residue management practice came out of studies[7,8,9,10,11,12,13] that did not take K leaching into account.

Potassium deficiency can be severe, particularly in pineapple cultivation on peat soils where K loss through leaching can be high due to low clay and the absence of mineral matter[14,15]. Potassium fixation is almost absent in peat soils[16] and despite their high cation exchange capacity, these soils do not readily adsorb exchangeable K[16]; a substantial amount of the total available K is always present in the soil solution and is hence strongly mobile and prone to leaching[16].

There is the need to quantify the total amount of K taken up by the plant (summation of K in roots, stem, leaves, peduncle, fruit, and crown), remaining in the soil after cultivation, and losses through leaching from applied K fertilizers on peat soils under pineapple cultivation. This assessment will be useful in evaluating K requirements of pineapples on peat soils, thereby contributing to the reduction of environmental pollution by excessive and unbalanced K fertilizer uses. The economic significance of efficient K use to non-K fertilizer-producing countries such as Malaysia, whose fertilizer use and import bill are higher than N[17], cannot be overemphasized. The import bill for K fertilizer (2001) has been has estimated at US$129.74 million per year (2001)[17]. This study was carried out to determine applied K use efficiency under a conventionally recommended fertilization regime in pineapple cultivation with residues removal.

## MATERIALS AND METHODS

The study was conducted in a pineapple estate in Johor, Malaysia on Umbro Saprists peat soil. The area has an annual precipitation of about 2000 mm. Monthly minimum and maximum temperatures are of 23 and 31˚C, and relative humidity ranged from 70 to 90%/month. Two treatments were used. Treatment one was on plots with K fertilizer (KF) and treatment two was on plots without K fertilization (NKF). Each experimental plot size was 4 × 12 m, with 300 suckers of *cv* Gandul (the most commonly grown variety) planted in each plot. The experimental plots were laid out in a randomized, complete block design with four replications.

Potassium was applied as KCl (49.8% K) to the KF plots. K fertilization schedules and rates were adapted to practices in the estate. At 83 days after planting (DAP), K was applied at 89 kg ha$^{-1}$ K. Another 89 kg ha$^{-1}$ K was applied at 144 DAP. At 209 DAP, 188 kg ha$^{-1}$ K was applied to the KF plots and the same rate was also applied at 263 DAP (Table 1). Normal estate N and P fertilization programs were followed. All other plant management procedures and schedules of the estate were also followed.

**TABLE 1**
**The Usual N and P Fertilization Program of the Pineapple Estate**

| | Number of Days of Application After Planting | | | |
|---|---|---|---|---|
| Fertilizer rate (kg ha$^{-1}$) | 83 | 144 | 209 | 263 (Total) |
| N | 176 | 176 | 176 | 176 (704) |
| P | 11 | 11 | 7 | 7 (36) |

Before the start of the experiment, pineapple residues were manually removed from the study area by slashing, raking, and removing old plants of the previous crop. Before removing the crop residues, peat soil samples were taken at depths of 0–10, 10–25, and 25–45 cm using peat soil auger and analyzed for total K, exchangeable K, and soil solution K using the dry ash, double acid, and squeeze methods[18,19,20], and atomic absorption spectroscopy (AAS). Subsequent peat soil samplings were done 48, 83, 144, 263, 365, 417, and 446 DAP. These samples were also analyzed for K forms mentioned using the aforementioned procedures. Peat core samplers of 7.5 cm diameter were used to collect peat soil samples at the stated depths and standard procedures were used to determine the bulk density of the experimental plots, before crop residues removal.

At 466 DAP, fresh fruits were harvested from the experimental plots excluding guard rows. A day before harvesting, three plants were randomly selected from the plots, uprooted, and partitioned into roots, stem, leaves, peduncle, fruit, and crown. This partitioning was done to enable calculation of the total uptake of K by the plants. These parts were oven dried at 60˚C until constant weights were attained and dry weights determined. Dry ash method was used to extract K from these tissues and AAS used to determine K concentrations in the tissues. Based on the plant density, a simple proportion was used to quantify K uptake in the different parts of pineapple plant per hectare. The K uptake in the plant parts for the fertilized and unfertilized treatments was compared by T test using the Statistical Analysis System[21].

The K recovery was calculated according to the formula of[22]:

$$\% \text{ fertilizer nutrient recovery} = (\text{TNF}) - (\text{TNU})/\text{R} \times 100$$

where TNF = total nutrient uptake from fertilized plots, TNU = total nutrient uptake from unfertilized plots, and R = rate of fertilizer nutrient applied.

## RESULTS AND DISCUSSION

The experimental plots did not significantly differ in their initial K forms (total, exchangeable, and soil solution). The bulk density of peat soils depends on the amount of compaction, the botanical composition of the materials, their degree of decomposition, and the mineral and moisture contents at the time of sampling[16]. Bulk densities at the depths of 0–10, 10–25, and 25–45 cm were 0.16, 0.23 and 0.13 g $cm^{-3}$, and were typical of Umbro Saprists peat soil[16]. But the relatively high bulk densities at the depths of 0–10 and 10–25 cm may be due to cultivation and compaction, subsidence of peat soil, of the surface layers on drainage[16]. The fact that the pineapple estate has been under use for about 36 years and has good drainage systems supports this observation. The lower bulk density at the depth of 25–45 cm may be due to partial decomposition of plant materials. Observation has shown that the bulk density in most tropical peat soils is higher at the surface layers than the subsurface layers as the former surfaces tend be more sapric (full decomposition) than the latter layers[16]. This has been associated with climate, height of water table, and oxidation[16]. Perhaps materials at the depth of 25–45 cm may have been lignified.

The distribution of K in the pineapple plant is presented in Table 2. There was no significant difference in the K uptake for roots, crown, and peduncle regardless of treatment difference, but this uptake was significantly different for stem, leaves, and fruit of the fertilized plants than the unfertilized plants. There was also significant difference across treatments (Table 2).

The total K uptake for KF and NKF were 498.84 and 342.50 kg $ha^{-1}$ (average across treatments), and with a total K rate of 554 kg $ha^{-1}$, K recovery was calculated (using the stated formula in materials and methods) to be only 28.22%. Not discounting the contribution of diffusion to this recovery[23,24], probably without the rooting system of pineapple, this recovery would have been lower. During growth, it is known that the adventitious roots of pineapples form a short and compact system at the stem base, with numerous strong roots and limited branching. However, under ideal conditions, the soil root system could spread up to 1–2 m laterally and 0.85 m in depth[25]. It therefore could be that unlike in mineral soils, the roots of pineapple in the peat soil were not very restricted or confined to the tilled area because of the relatively low bulk density. This might have allowed the roots under the fertilized condition to access larger volume of water and plants nutrients than those under the unfertilized condition. In addition, increased K diffusion gradient under the fertilized condition might have also facilitated the uptake of K in the fertilized plots[23,24].

**TABLE 2**
**Potassium Distribution in Pineapple Plant Parts**

| Parts | KF (kg ha$^{-1}$) | NKF (kg ha$^{-1}$) |
|---|---|---|
| Roots | 0.32[a] | 0.29[a] |
| Crown | 10.99[a] | 11.72[a] |
| Peduncle | 22.42[a] | 23.47[a] |
| Stem | 136.92[a] | 78.53[b] |
| Leaves | 139.23[a] | 87.61[b] |
| Fruit | 188.96[a] | 140.88[b] |
| Total | 498.84 [a] | 342.50[b] |

*Note:* Same alphabet within rows indicates no significant difference between treatment means using T test at $p$ = 0.05.

There was a sharp decrease of soil total K, exchangeable K, and soil solution K at 0–10 cm, after 263 DAP for plots with K fertilizer (Table 3). This decline continued until the end of the study such that the three different forms of soil K at the end of the study were generally lower than those before the study. There was, however, no evidence of significant accumulation of these K forms at depths of 10–25 and 25–45 cm. There seemed to be no corresponding significant accumulation of the K forms at deeper depths. Potassiaum concentrations throughout the study period were generally lower or equal to their initial status in the soil, indicating leaching. A comparison between the initial concentrations of total (Table 4), exchangeable (Table 5), soil solution (Table 6), and those at the end of the study showed that the latter concentrations were generally lower than the former. These observations suggest that the low K recovery of K could be attributed to leaching. Due to the low clay[14] and absence of mineral matter[15], K fixation that is noticeable in mineral soils is almost absent in peat soils. Although the cation exchange capacity of these soils is high, exchangeable K is not readily adsorbed. Other studies have shown that despite the high exchange capacity values (150–200 cmol [+] kg$^{-1}$ of organic soil), low mineral content of organic soils facilitated loss of applied fertilizer K remaining in the soil[18].

## CONCLUSION

Recovery of K in pineapple cultivation on tropical peat soil is low (28.22%) partly due to leaching loss. This loss through leaching needs to be considered in fertilizer recommendations for efficient recovery of soil applied K.

**TABLE 3**
**Concentrations of Three Different Forms of Soil K at Different Stages of Sampling for Three Different Depths**

| | **Stage of Sampling (DAP)** | | | | | | |
|---|---|---|---|---|---|---|---|
| | **0*** | **48** | **144** | **263** | **365** | **417** | **466** |
| | | | | **Total K (mg kg$^{-1}$)** | | | |
| | | | | 0–10 cm | | | |
| KF | 883[a] | 475[a] | 1450[a] | 2900[a] | 550[a] | 375[a] | 273[a] |
| NKF | 883[a] | 467[a] | 450[b] | 300[b] | 225[b] | 325[a] | 305[a] |
| | | | | 10–25 cm | | | |
| KF | 575[a] | 400[a] | 600[a] | 525[a] | 475[a] | 300[a] | 167[a] |
| NKF | 675[a] | 325[a] | 500[a] | 225[b] | 225[b] | 175[a] | 143[a] |
| | | | | 25–45 cm | | | |
| KF | 550[a] | 275[a] | 350[a] | 750[a] | 300[a] | 300[a] | 240[a] |
| NKF | 633[a] | 350[a] | 400[a] | 250[b] | 275[a] | 250[a] | 195[a] |
| | | | | **Exchangeable K (mg kg$^{-1}$)** | | | |
| | | | | 0–10 cm | | | |
| KF | 423[a] | 503[a] | 880[a] | 3330[a] | 538[a] | 260[a] | 290[a] |
| NKF | 540[a] | 513[a] | 408[b] | 320[b] | 147[b] | 260[a] | 278[a] |
| | | | | 10–25 cm | | | |
| KF | 460[a] | 468[a] | 635[a] | 698[a] | 623[a] | 360[a] | 330[a] |
| NKF | 417[a] | 535[a] | 623[a] | 343[b] | 238[b] | 215[a] | 155[a] |
| | | | | 25–45 cm | | | |
| KF | 423[a] | 453[a] | 445[a] | 450[a] | 395[a] | 383[a] | 383[a] |
| NKF | 383[a] | 447[a] | 430[a] | 383[a] | 393[a] | 300[a] | 245[b] |
| | | | | **Solution K (mg kg$^{-1}$)** | | | |
| | | | | 0–10 cm | | | |
| KF | 173[a] | 180[a] | 287[a] | 1593[a] | 320[a] | 313[a] | 98[a] |
| NKF | 182[a] | 148[a] | 67[b] | 94[b] | 68[b] | 160[b] | 93[a] |
| | | | | 10–25 cm | | | |
| KF | 89[a] | 60[a] | 102[a] | 173[a] | 160[a] | 175[a] | 100[a] |
| NKF | 95[a] | 68[a] | 82[a] | 36[b] | 53[b] | 85[a] | 33[a] |
| | | | | 25–45 cm | | | |
| KF | 114[a] | 58[a] | 54[a] | 77[a] | 130[a] | 210[a] | 70[a] |
| NKF | 89[a] | 68[a] | 41[a] | 56[a] | 80[a] | 150[a] | 48[a] |

* Before planting.

*Note:* Same alphabet within columns indicates no significant difference between means using T test at $p = 0.05$.

**TABLE 4**
**Comparison Between Soil Total K Before and After the Study**

| Treatment | K (mg kg$^{-1}$) | |
|---|---|---|
| | Before study | After study |
| | 0–10 cm | |
| KF | 883[a] | 273[b] |
| NKF | 883[a] | 305[b] |
| | 10–25 cm | |
| KF | 575[a] | 167[b] |
| NKF | 675[a] | 143[b] |
| | 25–45 cm | |
| KF | 633[a] | 143[b] |
| NKF | 550[a] | 240[b] |

*Note:* Same alphabet within rows indicates no significant difference between means before and after study using T test at $p$ = 0.05.

**TABLE 5**
**Comparison Between Soil Exchangeable K Before and After the Study**

| Treatment | K (mg kg$^{-1}$) | |
|---|---|---|
| | Before study | After study |
| | 0–10 cm | |
| KF | 423[a] | 290[a] |
| NKF | 540[a] | 278[b] |
| | 10–25 cm | |
| KF | 460[a] | 330[a] |
| NKF | 417[a] | 155[b] |
| | 25–45 cm | |
| KF | 423[a] | 383[a] |
| NKF | 383[a] | 245[b] |

*Note:* Same alphabet within rows indicates no significant difference between means before and after study using T test at $p$ = 0.05.

**TABLE 6**
**Comparison Between Soil Solution K Before and After the Study**

| Treatment | K (mg kg$^{-1}$) | |
|---|---|---|
| | Before study | After study |
| | 0–10 cm | |
| KF | 173[a] | 98[b] |
| NKF | 182[a] | 93[b] |
| | 10–25 cm | |
| KF | 89[a] | 100[a] |
| NKF | 95[a] | 33[b] |
| | 25–45 cm | |
| KF | 114[a] | 70[a] |
| NKF | 89 [a] | 48[a] |

*Note:* Same alphabet within rows indicates no significant difference between means before and after study using T test at $p$ = 0.05.
